# Preoperative molecular testing in thyroid nodules with Bethesda VI cytology: Clinical experience and review of the literature

**DOI:** 10.1002/dc.24637

**Published:** 2020-10-14

**Authors:** Emmanuel Labourier, Thomas J. Fahey

**Affiliations:** ^1^ Consultant Austin Texas USA; ^2^ Department of Surgery New York Presbyterian Hospital, Weill Cornell Medicine New York New York USA

**Keywords:** BRAF, miRNA, molecular testing, risk assessment, thyroid nodule

## Abstract

Risk assessment is critical to determine the timing of elective surgeries and preserve valuable resources in time of pandemic. This study was undertaken to better understand the potential value of molecular testing to risk‐stratify thyroid nodules with malignant cytology (Bethesda VI). Systematic review of the literature contributed 21 studies representing 2036 preoperative specimens. The BRAF p.V600E substitution was detected in 46% to 90% of cases with a pooled positivity rate of 70% (95% confidence intervals: 64%‐76%). None of the studies used comprehensive oncogene panels. Retrospective analysis of 531 clinical specimens evaluated with the next‐generation sequencing ThyGeNEXT Thyroid Oncogene Panel identified a total of 436 gene alterations. BRAF mutation rate was 64% in specimens tested as part of standard clinical care and 75% in specimens from cross‐sectional research studies (*P* = .022). Testing for additional actionable gene alterations such as TERT promoter mutations or RET and NTRK gene rearrangements further increased the diagnostic yield to 78%‐85% and up to 95% when including the ThyraMIR Thyroid miRNA Classifier. These data support the role of molecular cytopathology in surgical and therapeutic decision‐making and warrant additional studies.

## 1 INTRODUCTION

Multiple surgical societies have issued recommendations for a safe and responsible return to practice amid the COVID‐19 pandemic.^1^, ^2^, ^3^ As elective surgeries resume, surgeons face a major backlog of cases and must prioritize patients based on individual's risk, local and state requirements and available resources. Preoperative risk stratification of thyroid nodules typically involves a combination of blood work, imaging and fine needle aspiration biopsy (FNAB), as well as molecular testing for nodules with indeterminate cytology.^4^, ^5^, ^6^ These testing modalities are also offered clinically to aid surgical and therapeutic decisions for advanced or metastatic thyroid cancers and for nodules with high‐risk cytopathologic features. Yet, little information is available on the type and distribution of gene alterations present in FNAB positive for malignancy (Bethesda category VI). To address this point, we conducted a systematic review of the literature and analyzed molecular data generated in two distinct sets of preoperative FNAB: (1) Representative clinical cases submitted to a CLIA‐certified laboratory for evaluation with the next‐generation sequencing ThyGeNEXT Thyroid Oncogene Panel; and (2) Cross‐sectional cohort of cases previously collected and tested during the development and validation of the ThyraMIR Thyroid miRNA Classifier. The data underscore the value of comprehensive, actionable molecular results to further the risk assessment of thyroid nodules with malignant cytology and inform clinical decision‐making.

## 2 MATERIALS AND METHODS

The biomedical literature database from the National Center for Biotechnology Information (https://pubmed.ncbi.nlm.nih.gov/) was searched to identify relevant studies published after 2010 and reporting more than 5 positive cases. Studies limited to RNA biomarkers were not included in the analysis. Retrospective review of cases previously tested as part of standard clinical care in Interpace Biosciences' laboratory or as part of clinical research studies was performed exclusively on deidentified molecular data. The analyses did not involve any clinical data or protected health information that could be linked to individual subjects and did not constitute human subjects research as defined in 45 CFR 46.102. Pooled rates and I^2^ heterogeneity tests were calculated using fixed or random effect models and the Cochran Q statistic. Proportions were compared using Pearson chi‐square tests.

## 3 RESULTS

**FIGURE 1:**
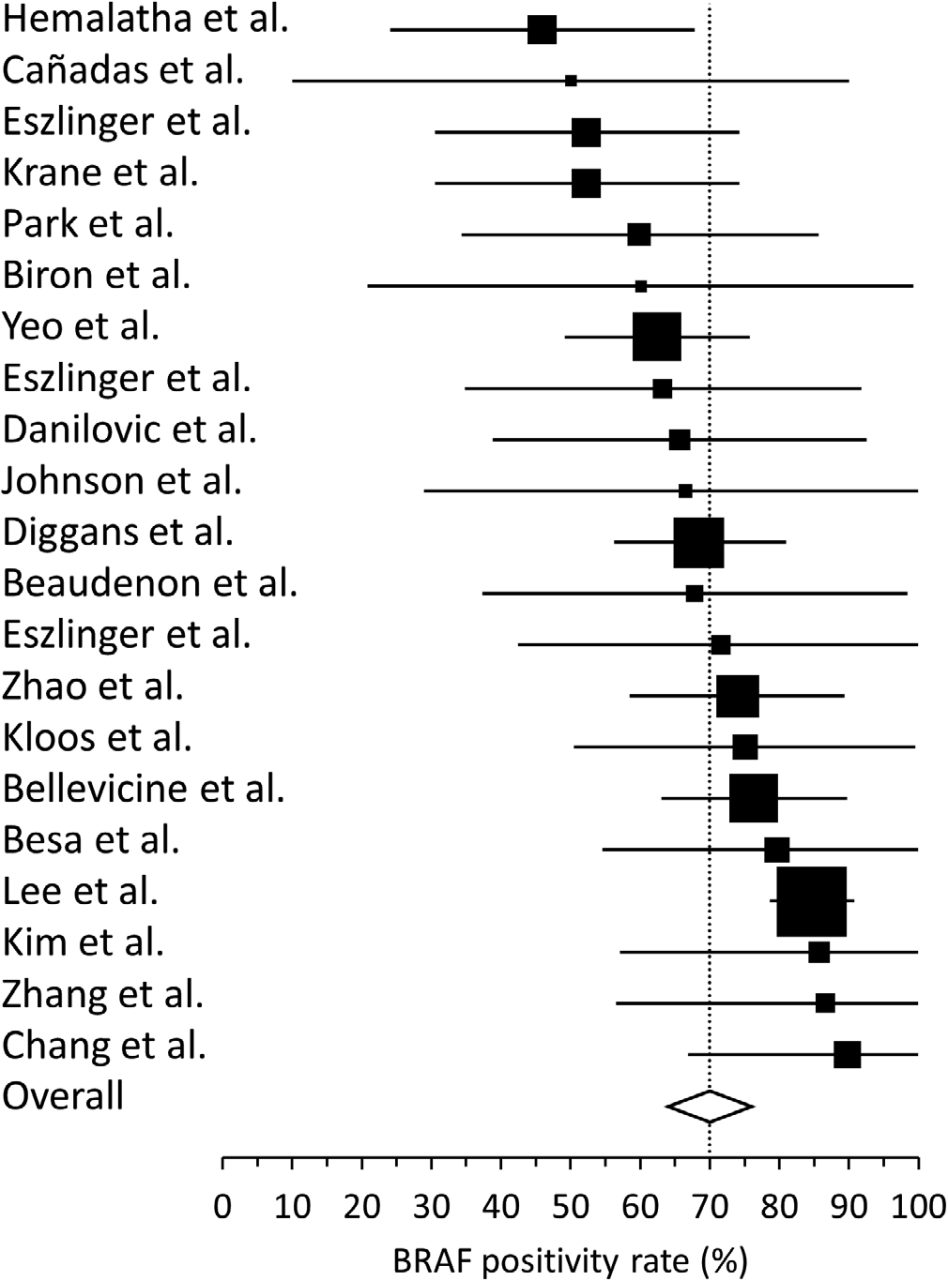
Forest plot for BRAF p.V600E positivity rates reported in 21 peer‐reviewed publications.^7^, ^8^, ^9^, ^10^, ^11^, ^12^, ^13^, ^14^, ^15^, ^16^, ^17^, ^18^, ^19^, ^20^, ^21^, ^22^, ^23^, ^24^, ^25^, ^26^, ^27^ Black boxes represent the relative weight of each study, whiskers represent the 95% confidence intervals for each reported BRAF rate and the diamond represents the calculated pooled rate assuming a random effect model (70% pooled rate, 95% confidence intervals: 64%‐76%, I^2^ = 0%)

Review of the literature identified 21 studies from 12 countries involving n = 2036 preoperative thyroid specimens with malignant/Bethesda VI cytology (Table 1).^7^, ^8^, ^9^, ^10^, ^11^, ^12^, ^13^, ^14^, ^15^, ^16^, ^17^, ^18^, ^19^, ^20^, ^21^, ^22^, ^23^, ^24^, ^25^, ^26^, ^27^ BRAF p.V600E (c.1799T>A) positivity rate ranged from 46% to 90% with a median rate of 68%. Heterogeneity across studies was moderate. The pooled BRAF rate was 75% (95% confidence intervals: 71% to 78%, I^2^ = 46%) for a fixed effect model and 70% (95% confidence intervals: 64% to 76%, I^2^ = 0%) for a random effect model (Figure 1). The numbers of positive cases and reporting studies were too low to calculate pooled statistics for other gene alterations. Only six studies (n = 339 cases) used multi‐gene panels interrogating various combinations of BRAF, RAS, RET and PAX8 gene alterations. Four of these studies were from the same group.^9^, ^10^, ^14^, ^19^ RET‐PTC rearrangements were detected in four studies (n = 13 cases, 3.8%), RAS mutations in three studies (n = 6 cases, 1.8%) and PAX‐PPARG in a single study (n = 2 cases, 0.6%).

**TABLE 1:** Rates of BRAF p.V600E substitution in preoperative thyroid nodule specimens with malignant/Bethesda VI cytology reported in the literature^7^, ^8^, ^9^, ^10^, ^11^, ^12^, ^13^, ^14^, ^15^, ^16^, ^17^, ^18^, ^19^, ^20^, ^21^, ^22^, ^23^, ^24^, ^25^, ^26^, ^27^

Study	Year	Country	Method	Cases, No.	BRAF rate (%)
Hemalatha et al.^7^	2018	India	Sanger sequencing	37	46
Cañadas‐Garre et al.^8^	2012	Spain	PCR RFLP	12	50
Eszlinger et al.^a^ ^,^ ^9^	2014	Denmark	Pyrosequencing	42	52
Krane et al.^a^ ^,^ ^10^	2015	United States	Pyrosequencing	42	52
Park et al.^11^	2013	South Korea	Real‐time PCR	35	60
Biron et al.^12^	2018	Canada	Digital droplet PCR	15	60
Yeo et al.^13^	2012	South Korea	Pyrosequencing	136	63
Eszlinger et al.^a^ ^,^ ^14^	2015	Italy	Pyrosequencing	30	63
Danilovic et al.^15^	2014	Brazil	Real‐time PCR	35	66
Johnson et al.^16^	2014	United Kingdom	PCR melting curve	18	67
Diggans et al.^17^	2015	United States	Real‐time PCR	172	69
Beaudenon et al.^a^ ^,^ ^18^	2014	United States	PCR hybridization	28	68
Eszlinger et al.^a^ ^,^ ^19^	2017	Germany	Pyrosequencing	32	72
Zhao et al.^20^	2015	China	Sanger sequencing	119	74
Kloos et al.^21^	2013	United States	Real‐time PCR	48	75
Bellevicine et al.^a^ ^,^ ^22^	2020	Italy	Real‐time PCR	165	76
Beiša et al.^23^	2016	Lithuania	Real‐time PCR	49	80
Lee et al.^24^	2012	South Korea	MEMO sequencing	876	85
Kim et al.^25^	2018	South Korea	Real‐time PCR	41	85
Zhang et al.^26^	2015	China	Real‐time PCR	37	86
Chang et al.^27^	2012	South Korea	PCR melting curve	67	90

Abbreviations: PCR, polymerase chain reaction; RFLP, restriction fragment length polymorphism; MEMO, mutant enrichment with 3′‐modified oligonucleotide.

^a^ Studies evaluating BRAF, RAS, RET and PAX8 gene alterations.

Review of cases submitted for molecular testing as part of standard clinical care between April 2015 and June 2020 yielded 381 unique Bethesda VI specimens with molecular data (Table 2). One or several genetic alterations were detected in 78% (162/209) of the specimens tested with ThyGeNEXT and in 73% (125/172) of the specimens tested with previous versions of the test (*P* = .28). The exact nucleotide variations are described in Supplemental Table S1. Overall, 75% (287/381) of the specimens were positive by molecular testing, 270 for mutations in the BRAF, PIK3CA, RAS and/or TERT genes (94% of positive cases) and 17 for PAX8, NTRK or RET gene rearrangements (6% of positive cases). The most frequent alterations were BRAF p.V600E (64%) and TERT promoter mutations (11%).

**TABLE 2:** Distribution of molecular results in n = 381 CLIA specimens

Molecular result	Pre‐ThyGeNEXT	ThyGeNEXT	Combined
BRAF	111	120	231
PIK3CA	1	0	1
RAS	7	7	14
CCDC6‐RET	4	7	11
NCOA4‐RET	1	2	3
PAX8‐PPARG	1	0	1
BRAF + TERT^a^	n/a	14	14
PIK3CA + TERT^a^	n/a	1	1
RAS + TERT^a^	n/a	5	5
TERT^a^	n/a	4	4
ETV6‐NTRK^a^	n/a	1	1
TRIM24‐RET^a^	n/a	1	1
Negative	47	47	94
Total	172	209	381

^a^ Gene alteration not interrogated in previous versions of the ThyGeNEXT test.

**FIGURE 2:**
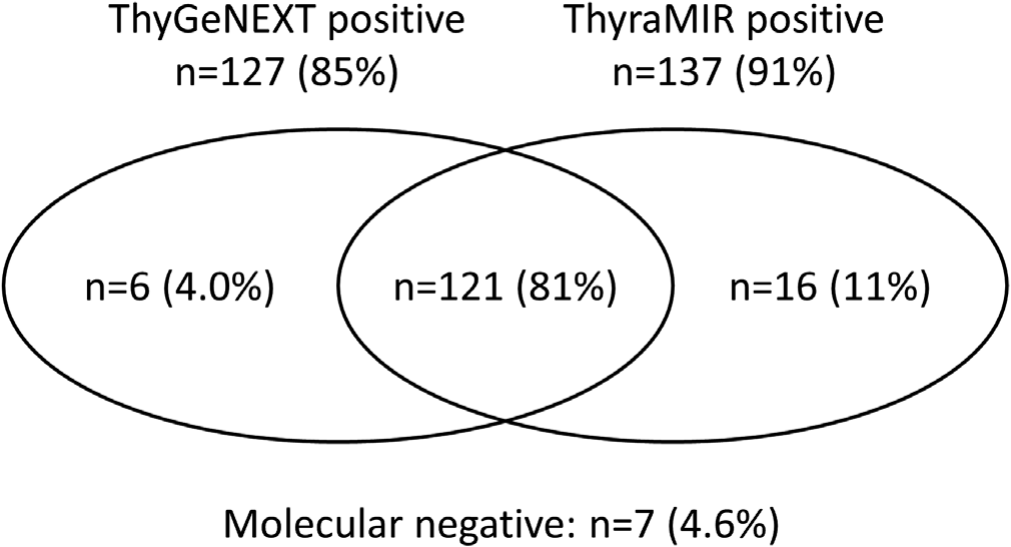
Venn diagram showing the relationship between ThyGeNEXT and ThyraMIR molecular results in n = 150 research specimens

Review of cases collected and tested as part of clinical research studies yielded 150 Bethesda VI specimens with molecular data (Table 3). There were 127 specimens (85%) positive for one or several gene alterations, 117 for mutations in the BRAF, RAS and/or TERT genes (92% of positive cases) and 10 for PAX8, NTRK or RET gene rearrangements (8% of positive cases). BRAF p.V600E was detected in 112 specimens (75% of all cases). The ThyraMIR test classified 137 specimens (91%) as high risk for malignancy, including the majority of mutation positive cases (121/127 or 95%) (Figure 2). Out of 23 mutation negative specimens, the miRNA classifier identified 16 cases (70%) as positive/high risk, further increasing the yield of molecular testing to 95% overall (143/150).

**TABLE 3:** Distribution of molecular results in n = 150 research specimens

Molecular result	Pre‐ThyGeNEXT	ThyGeNEXT	Combined
BRAF	88	22	110
RAS	5	0	5
CCDC6‐RET	5	1	6
NCOA4‐RET	1	0	1
PAX8‐PPARG	1	0	1
BRAF + TERT^a^	n/a	2	2
ETV6‐NTRK^a^	n/a	2	2
ThyraMIR high risk	13	3	16
Negative	5	2	7
Total	118	32	150

^a^ Gene alteration not interrogated in previous versions of the ThyGeNEXT test.

## 4 DISCUSSION

In our clinical experience analysis, a total of 436 gene alterations were identified in 414 of 531 preoperative specimens with malignant cytology. There were significant differences between the two cohorts and their respective collection schemes. The clinical research cohort was a representative cross‐sectional sampling of thyroid nodules at the time of FNAB diagnosis while the CLIA cohort consisted of more challenging cases that required molecular testing as part of their routine clinical management. Predictably, the positivity rate was lower among CLIA specimens relative to research specimens (75% vs 85%, *P* = .019). The majority of positive cases harbored a BRAF p.V600E substitution in both cohorts (67% overall), 64% for CLIA specimens and 75% for research specimens (*P* = .022). The second most frequent gene alteration was TERT promoter mutations (26/241 or 11%), highlighting the limitation of molecular strategies that only interrogate exome variants. For example, the Afirma Xpression Atlas, based on transcriptome RNA sequencing, has a reported false negative rate of 26% relative to targeted DNA sequencing and cannot assess variants outside the fraction of exonic sequences that are actually transcribed into mRNA.^28^

The BRAF positivity rates observed in the two clinical cohorts were consistent with the 70% pooled rate estimated from 21 published studies (95% confidence intervals: 64% to 76%). The use of different molecular methods, reagents and protocols probably contributed to the broad range of reported rates (46% to 90%). Methods with poor analytical sensitivity, for example, Sanger sequencing, or with clinical cutoffs set at high percent variant, are expected to have a lower detection rate. In one of the reviewed studies, Lee et al^24^ evaluated 876 Bethesda VI specimens using three distinct molecular methods and reported BRAF detection rates of 63%, 79% and 85% for methods with an increasing analytical sensitivity of 20%, 2% and 0.1% variant, respectively. Another parameter likely contributing to the heterogeneity of BRAF rates was regional variations in the prevalence of BRAF mutation and/or papillary thyroid carcinoma. For example, three out of the five studies with the highest reported BRAF rates (80% to 90%) were conducted in South Korea, a region where BRAF p.V600E is particularly prevalent.^24^, ^25^, ^27^ In a series of 200 resected conventional papillary carcinomas from South Korea, Seo et al^29^ showed that 93% of the surgical specimens were positive for BRAF p.V600E and that 96% of these cases could be detected in the corresponding malignant FNAB.

The high frequency of oncogenic BRAF mutations in preoperative thyroid FNAB has important clinical implications. Multiple studies have shown that BRAF p.V600E correlates with aggressive features of thyroid carcinoma such as extrathyroidal extensions, vascular invasion, larger nodule size, advanced staging, lymph node metastasis and recurrence.^4^, ^5^, ^6^ A large multicenter study recently demonstrated that BRAF is associated with mortality in older patients, independently of other clinicopathologic risk factors.^30^ Other studies have suggested that knowledge of the BRAF mutational status may be useful to guide the extent of thyroid surgery.^31^, ^32^ However, the association of BRAF p.V600E with worse prognosis independent of other risk factors remains debated and requires additional investigation. Testing with a comprehensive oncogene panel that includes relevant and actionable gene alterations such as TERT promoter mutations or RET and NTRK gene rearrangements can also aid surgical decision‐making and speed up individualized patient care. Molecular insights may change a patient's risk profile when multiple markers are detected in the same nodule, identify carcinomas that are refractory to radioactive iodine treatment or facilitate the selection of targeted therapies.^6^, ^33^, ^34^, ^35^ Combination testing with a miRNA expression classifier may further raise the risk profile of nodules positive for weaker driver mutations such as RAS and increase the positive diagnostic yield up to 95%. Because of their unique biology, miRNAs are practical surrogate markers to identify altered oncogenic pathways in mutation negative cells and in heterogenous thyroid nodules where only a small fraction of tumor cells may carry a somatically acquired gene alteration.^36^, ^37^

In summary, our systematic review of the literature indicates that 70% to 75% of FNAB with malignant/Bethesda VI cytology are expected to be positive for the oncogenic BRAF p.V600E substitution. The majority of these studies (71%) assessed only BRAF mutational status. Our analysis of 531 representative clinical specimens is the first to report the potential value of a comprehensive oncogene panel combined with a miRNA expression classifier. Additional work is required to fully assess the role of molecular testing for the preoperative risk stratification of malignant FNAB, clinical decision‐making, timing of surgery and optimal utilization of valuable healthcare resources.

## CONFLICT OF INTEREST

E.L. is a consultant for Interpace Biosciences Inc.

## Supporting information


**Table S1** Distribution of DNA mutations in n = 270 CLIA specimens positive with the ThyGeNEXT Thyroid Oncogene Panel.

## Data Availability

The data that support the findings of this study are available from the corresponding author upon reasonable request.
