# Supplementary material for: Preoperative molecular testing in thyroid nodules with Bethesda VI cytology: Clinical experience and review of the literature
Source: Diagn Cytopathol. 2020 Oct 14;49(4):E175–80. doi: 10.1002/dc.24637 (PMC7983887; doi:10.1002/dc.24637)
Supplement: Supplementary file 1 — Table S1 Distribution of DNA mutations in n = 270 CLIA specimens positive with the ThyGeNEXT Thyroid Oncogene Panel. [file DC-49-E175-s001.docx]

**Supplemental Table 1.** Distribution of DNA mutations in n=270 CLIA specimens positive with the ThyGeNEXT® Thyroid Oncogene Panel.

| Mutation | Cases, No. |
| --- | --- |
| BRAF c.1799T>A | 231 |
| BRAF c.1799T>A + TERT c.-124C>T | 12 |
| BRAF c.1799T>A + TERT c.-146C>T | 2 |
| HRAS c.37G>C | 1 |
| HRAS c.181C>A | 1 |
| HRAS c.182A>G + TERT c.-124C>T | 2 |
| KRAS c.35G>A | 3 |
| KRAS c.35G>A + TERT c.-124C>T | 1 |
| KRAS c.35G>T | 1 |
| KRAS c.35G>T + TERT c.-124C>T | 1 |
| KRAS c.38G>A | 1 |
| KRAS c.182A>G | 1 |
| NRAS c.35G>T + TERT c.-124C>T | 1 |
| NRAS c.181C>A | 3 |
| NRAS c.182A>G | 3 |
| PIK3CA c.1624G>A | 1 |
| PIK3CA c.163G>A + TERT c.-124C>T | 1 |
| TERT c.-124C>T | 3 |
| TERT c.-146C>T | 1 |
